# Compensation claims for chiropractic in Denmark 2013–2022

**DOI:** 10.1186/s12998-026-00627-1

**Published:** 2026-02-24

**Authors:** Amalie Horstmann Nøddeskou-Fink, Martin Skoumann Jørgensen, Jan Hartvigsen, Henrik Wulff Christensen, Mette Jensen Stochkendahl

**Affiliations:** 1 Center for Muscle and Joint Health, Department of Sport Science and Clinical Biomechanics, University of Southern Denmark, Odense, Denmark; 2 Chiropractic Knowledge Hub, University of Southern Denmark, Odense, Denmark; 3 Private Practice of Chiropractic, KiroPraktisk, Ryttervej 49, 5700 Svendborg, Denmark; 4 Private Practice of Chiropractic, Kiropraktorhuset Næstved, Jernbanegade 17, 4700 Næstved, Denmark

**Keywords:** Chiropractic, Manual therapy, Compensation claims, Adverse events, Cervical artery dissection, Side effects, Injuries, Healthcare, Primary care

## Abstract

**Background:**

Injuries sustained during healthcare consultations are a significant concern, and compensation claims relating to injuries in health systems are increasing. Extensive research has addressed injuries in the secondary sector, whereas knowledge about injuries sustained in primary care remains sparse. This retrospective register-based study aimed to describe compensation claims involving chiropractors in Denmark between 2013 and 2022.

**Methods:**

All claims related to chiropractors from 2013 to 2022 were accessed in the Danish Patient Compensation Association Register and analyzed using the Healthcare Complaints Analysis Tool. Data on patient characteristics, injuries, processing time, decisions, appeals, and financial compensation were collected. Claims were categorized as relating to clinical, management, or patient-clinician relationship, alongside nine symptom-based injury classifications. Data relating to cervical artery dissection were examined separately and in greater detail, including information on presenting symptoms, International Classification of Primary Care, Second Edition code recorded by the chiropractor, treatment modalities used, time from treatment to onset of symptoms, and type of vascular injury subsequently diagnosed. Descriptive statistics summarized findings.

**Results:**

A total of 535 chiropractor-related claims were identified, with 519 included for analysis. The number of claims per 100,000 consultations increased from 1.03 in 2013 to 3.57 in 2022. Most claims (84%) concerned treatment outcomes and side effects, primarily worsening of symptoms (23%) or delayed referral (23%). Of the 519 claims, only 32 (6%) were approved for compensation. Cervical artery dissection-related claims had the highest approval rate within category (29%; ~ 0.7 approved claims per million consultations) and accounted for 94% of total financial compensation (14 approved claims, 3,025,000 €).

**Conclusion:**

Compensation claims related to chiropractic care in Denmark increased between 2013 and 2022, but approval rates remained low. Most claims concerned dissatisfaction with treatment outcome or worsening of symptoms. Cervical artery dissection-related claims had the highest approval rate and accounted for the highest compensation. When approved, they were compensated based on the fairness rule stating that the outcome could neither have been predicted nor expected from patients’ individual cases. Better communication between patients and chiropractors about expectations for treatment, natural course of conditions, and expected reactions to treatment will likely reduce the number of claims.

**Supplementary Information:**

The online version contains supplementary material available at 10.1186/s12998-026-00627-1.

## Background

Adverse events in which patients’ illnesses or health conditions worsen due to tests or treatments within healthcare systems constitute a significant global problem [1–4]. While such adverse events may have serious or fatal consequences in hospital settings, [2–5] they are often milder and transient in the primary healthcare sector [6–8]. These include pain or discomfort, side effects of medication, worsening of symptoms, or delayed diagnosis or referrals [1, 6–9]. In 2023, nearly 410,000 adverse events were reported to the Danish Patient Safety Database, encompassing incidents from both hospital and primary healthcare settings [10]. These events covered a broad spectrum, including falls related to the physical environment, patient misidentification, healthcare-associated infections, and errors involving insufficient or incorrect oxygen administration [10].

In 2014, Jevne et al. [9] investigated injury compensation claims filed against Danish and Norwegian chiropractors between 2004 and 2012, and found that chiropractors, on average, received one compensation claim per 100,000 consultations. Jevne et al. [9] found that many of the claims against chiropractors related to insufficient patient information suggested that many could have been avoided if the chiropractor had informed the patient about the natural course of their condition and common benign side effects of manual treatment (such as local discomfort, radiating discomfort, tiredness, headache, or dizziness) [9, 11].

A particular concern relating specifically to manual treatment of the neck is cervical artery dissections (CAD) resulting in cerebrovascular events and stroke, which has been linked to chiropractic care [12–16]. Although no causal relationship between manual therapy and CAD has been established [14, 17, 18] and even though the risk of cerebrovascular events after a consultation with a chiropractor is similar to that following a consultation with a general practitioner [19], the issue remains a subject of concern and debate [20, 21].

Within the Danish tax–funded, public healthcare system, the Danish Patient Compensation Association [22] determines whether patients qualify for financial compensation for injuries (physical, mental, or financial) sustained as a result of their interaction with the healthcare system [23]. Patients are entitled to compensation in case of delayed, incorrect, or insufficient treatment, incorrect diagnosis, post-surgical complications, side effects from medication, or defective medical equipment. Compensation is paid as lump sum, with the amount being determined by several factors, including loss of work capacity and temporary loss of income. Costs of medical care, sickness absence, or premature retirement are covered by the Danish welfare system and therefore not included when considering the level of compensation. The Danish Patient Compensation Association functions under a no-fault system (similar systems are found in Sweden and also New Zealand), where claims filed do not result in direct consequences for the healthcare professional involved [24–26]. In Denmark, healthcare professionals by law have an obligation to inform the patient about the compensation system, and to, if necessary, help the patient report any potential compensable injuries [27]. In addition to providing security and service for patients and healthcare personnel, the Danish Patient Compensation Association encourages the use of registered claims to help prevent future injuries. The claims provide insight into both the type of injuries and the patients’ views and experiences, which is essential in health learning systems for ensuring development and optimisation of patient safety [6, 7, 28].

From 2012 to 2022, the chiropractic profession in Denmark grew by approximately 34% (from 524 to 705 chiropractors) [29, 30] and continues to attract more patients each year [31]. In 2012, Danish chiropractors holding a provider number (and therefore practicing within the national reimbursement framework) did 1.9 million consultations [31]. This number rose to 2.0 million (3.6% increase) consultations in 2022 [31].

The overall aim of this retrospective study is to update and describe patient compensation claims concerning chiropractors in Denmark from 2013 to 2022.

Specific objectives are to:

- Report the number of patient compensation claims per year and per 100,000 consultations
- Describe patient compensation claims in terms of: types of claims, patient characteristics, claim processing time, approval rates, appeals, and level of financial compensation granted
- Provide a detailed description of claims involving CAD, including information on presenting symptoms, International Classification of Primary Care, Second Edition (ICPC-2) [32] diagnosis code recorded by the chiropractor, treatment modalities used, time from treatment to onset of symptoms, type of vascular injury, and level of financial compensation

## Method

### Study design

This study is a retrospective registry-based study using data from the Danish Patient Compensation Association’s national patient registry.

### Population and materials

The study population consists of patients who had visited a chiropractor and filed a claim relating to the visit(s) with the Danish Patient Compensation Association. We included all claims with a registration date in the Danish Patient Compensation Association system between January 1st, 2013, and December 31st, 2022. Claims were not included if they were registered outside the study period, did not involve a chiropractor, were withdrawn, or did not constitute a compensation claim.

The Danish Patient Compensation Association covers claims against all authorized Danish chiropractors. Approximately 90% of authorized Danish chiropractors hold a registered provider number and operate within the national reimbursement framework, providing reimbursement for the patients. The remaining 10% of chiropractors operate without national reimbursement and price regulation, but within the same public health legislation.

To determine whether a patient qualifies for compensation under the Danish Patient Compensation Association, at least one of three criteria/rules must be met: 1) *the specialist rule* stating that the injury could have been avoided if the patient had been treated according to the standard of an experienced specialist in the area, 2) *the fairness rule* stating that the patient should be awarded compensation if they—despite no mistakes or faults in procedures and treatments—are in worse condition than could be expected after encounter with healthcare system, and 3) *the medical equipment rule* stating that patients should be awarded compensation in the case of malfunction of medical equipment [23].

### Procedures, data extraction, and variables

Data were extracted from the registered claims using the *Healthcare Complaints Analysis Tool* (HCAT) [33–35]. HCAT is an internationally recognized data extraction and analytic tool that enables comparison across international healthcare systems [34, 36, 37]. HCAT focuses on the patient’s written account of their experience [34]. It is based on the tripartite taxonomy of patient complaints proposed by Reader et al., categorizing patient complaints into three domains: clinical, management, and relationship, subdivided into two or three problem categories with subcategories [36].

As HCAT is developed for hospital complaints rather than primary care compensation claims, we omitted variables specifically pertaining to hospitals (e.g., dates of hospitalization and discharge, inadequate hospital staffing, and poor food).

To enable comparison with the result from Jevne et al. [9] we extracted the same variables relating to complaint categories not covered by HCAT. Further variables around CAD were also extracted, including: symptoms prior to treatment, the time interval between treatment and the onset of symptoms, and the type of vascular injury diagnosed (Supplementary File 1 and 2).

Before the data extraction, two members of the research team (AHNF and MSJ) underwent a structured training program in HCAT, piloted and adjusted the data entry module (Epidata). To ensure inter-rater agreement, a random sample of 10% of the claims was drawn and data extracted. Discrepancies occurred in 6% of the recorded variables, corresponding to an inter-rater reliability of 94%. Discrepancies were discussed, corrected, and documented to ensure consistency in the data extraction of the remaining claims, which is expected to have further improved the study’s inter-rater reliability. The remaining claims were randomly and evenly distributed between the two authors for independent data extraction.

### Analysis

Statistics Denmark holds data on the yearly number of consultations with registered chiropractors working under the collective agreement [31, 38]. Claims were categorized into HCAT and Jevne's categories, which were tabulated, and approval rates and financial compensations were calculated for each category. We first calculated the total number of injuries and divided by the total number of consultations registered by Statistics Denmark between 2013 and 2022 (incl.), then did the same per year. Results are presented as the number of claims per 100,000 consultations. We calculated annual approval rates by dividing the number of approved claims by the number of claims made during a year. The mean claim processing time was calculated by summing the number of days between the claims’ submission dates and the decision dates, divided by the number of claims. Pending claims were not included in the calculation of approval rates or mean claim processing time.

All monetary values are presented in Euros (€), representing the value of the compensation in the year it was granted.

## Results

**Fig. 1:**
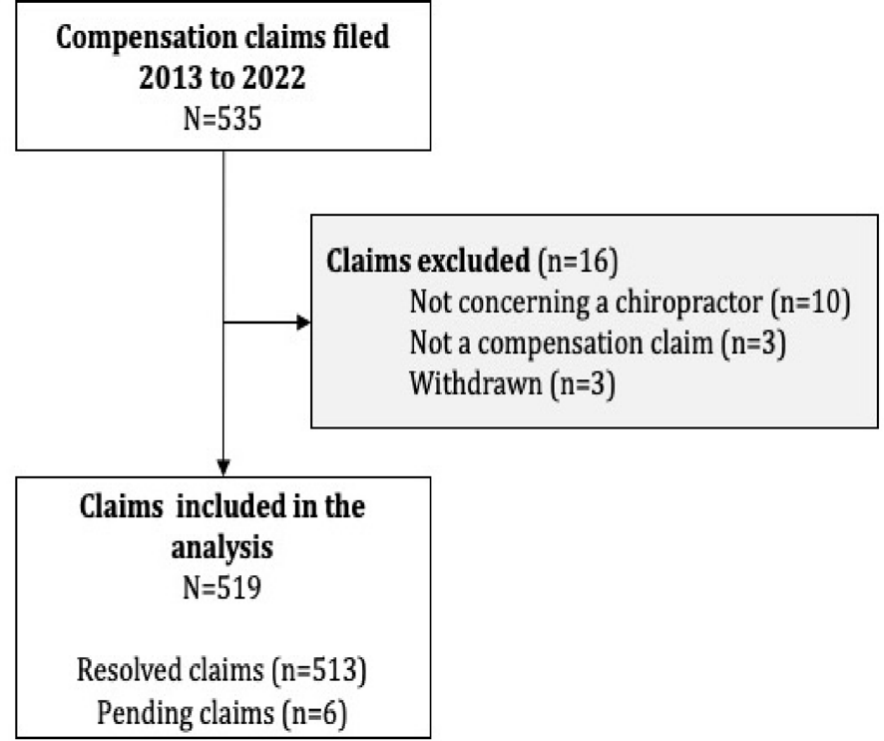
Flowchart of included compensation claims

Between 2013 and 2022, 535 compensation claims dealing with chiropractors were registered by the Danish Patient Compensation Association. Of these, 16 claims were excluded, leaving 519 claims included for analysis (Fig. 1). Six claims were pending at the time of analysis.

During the study period, 51.9 claims were filed per year on average. This corresponds to an average of 2.6 patient compensation claims per 100,000 consultations. The annual number of patient compensation claims per 100,000 consultations increased over the study period from 1.03 in 2013 to 3.57 in 2022 (Table 1).

**Table 1:** Overview of patient compensation claims involving a chiropractor

Year	Claims	Approved claims		Financial compensation	Percentage of total compensation	Annual number of claims per 100,000 consultations
	n	n	(%)	€	(%)	n
2013	20	5	(25.0)	1,551,420	(51.3)	1.03
2014	38	2	(5.3)	98,845	(3.3)	1.93
2015	44	2	(4.5)	9,480	(3.3)	2.17
2016	58	4	(6.9)	585,721	(19.4)	2.78
2017	58	3	(5.2)	215,709	(7.1)	2.81
2018	52	2	(3.8)	18,298	(0.6)	2.54
2019	67	4	(6.0)	23,757	(0.8)	3.19
2020	53	3	(5.7)	417,370	(13.8)	2.70
2021	58	3	(5.3)	23,362	(0.8)	2.72
2022	71	4	(6.1)	80,653	(2.7)	3.57
Total	519	32	(6.2)	3,024,616	(100)	2.54

^*^By the time of analysis, 6 claims were pending (5 from 2022 and 1 from 2021) and therefore not included

Of the 519 claims, 299 (58%) involved a female patient, and most claims involved patients aged 30–59 years, accounting for 330 (64%) of the claims.

A total of 692 subproblems were reported, with 144 (28%) of claims mentioning more than one subproblem. Six claims were coded as having more than three subproblems. Of the 692 recorded subproblems, 493 (71%) related to the clinical domain *Quality*. Among these, most (88%) were categorized as relating to the outcome of treatment or side effects, totaling 436 (84%) of the 519 claims. Only 81 (12%) of all reported subproblems were classified as *High* severity level (Table 2).

**Table 2:** Distribution of HCAT problem categories, subproblems, and severity levels

Problem categories and subproblems		Total*		Level of severity					
				Low		Medium		High	
		n	(%)	n	(%)	n	(%)	n	(%)
Clinical	Quality	493	(71.2)	226	(45.8)	204	(41.4)	63	(12.8)
	Outcomes and side effects	436	(88.4)						
	Rough handling and discomfort	45	(9.1)						
	Examination and monitoring	8	(1.6)						
	Making and following care plans	4	(0.8)						
	Safety	133	(19.2)	29	(21.8)	87	(65.4)	17	(12.8)
	Error—diagnosis (missed, delayed, incorrect)	128	(96.2)						
	Error—general	1	(0.8)						
	Failure to respond	2	(1.5)						
	Clinician skills	2	(1.5)						
Relationship	Listening	42	(6.1)	23	(54.8)	19	(45.2)	0	(0.0)
	Token listening	27	(64.3)						
	Ignoring patients	10	(23.8)						
	Dismissing patients	5	(11.9)						
	Respect and patient rights	11	(1.6)	3	(27.3)	9	(81.8)	0	(0.0)
	Consent	7	(63.6)						
	Disrespect	4	(36.4)						
	Communication	10	(1.4)	5	(50.0)	5	(50.0)	0	(0.0)
	Absent communication	6	(60.0)						
	Insufficient involvement of relatives	1	(10.0)						
	Incorrect communication	3	(30.0)						
Management	Enviroment	2	(0.3)	1	(50.0)	0	(0.0)	1	(50.0)
	Equipment	2	(100.0)						
	Institutional processes	1	(0.1)						
	Clinical prioritisation	1	(100.0)	1	(100.0)	0	(0.0)	0	(0.0)

^*^Totals are presented both within problem categories and within subproblems

When categorizing according to Jevne’s [9] complaint categories, 211 (41%) claims were due to the patient experiencing worsening of symptoms, new symptoms, or a new injury following treatment. Together, worsening of symptoms and delayed referral were the two most common reasons for filing a claim, each accounting for 119 (23%) claims (Table 3). All approved CAD claims were approved based on the fairness rule. Of the pending claims, three were in the CAD category, two in newly developed symptoms, and one in alleged disc herniation.

**Table 3:** Distribution of claims according to symptom categories

Category	Total number of claims		Approved claims	Percentage of claims approved within the category*	Total financial compensation paid	Percentage of total compensation
	n	(%)	n	(%)	€	(%)
Worsening of symptoms	119	(22.9)	0	(0)	0	(0,0)
Disc herniation	74	(14.3)	2	(2.7)	26,197	(0,9)
Delayed referral	119	(22.9)	5	(4.2)	17,669	(0,6)
Newly developed symptoms/injury following treatment	92	(17.7)	7	(7.8)	100,428	(3,3)
Fractures (not including ribs)	22	(4.2)	2	(9.1)	18,298	(0,6)
Cervical artery dissection (CAD)	51	(9.8)	14	(29.2)	2,851,309	(94,3)
Rib injury (including fractures)	28	(5.4)	1	(3.6)	4,552	(0,2)
Accidents	5	(1.0)	1	(20.0)	6,261	(0,2)
Miscellaneous	9	(1.7)	0	(0)	0	(0)
Total	519	(100)	32	(6.2)	3,024,616	(100)

^*^The percentage of claims approved is calculated based on claims resolved (n = 513)

Claim processing time varied according to the complexity of the claim with a median of 22 days (range 2–1175 days) from submission date to decision. If the decision was appealed or reopened, the duration was longer.

Of the 519 included claims, 32 (6%) were approved with financial compensation. Four claims (0.8%) were approved according to the *specialist rule*; 22 claims (4.2%) were approved according to the *fairness rule*, and 6 claims according to the *medical equipment rule* (1.2%).

The annual approval rate ranged from 4 to 7% over the period, excluding 2013, when 25% of the claims were approved. There were 29 claims rejected because the compensation amount was below the compensation threshold (yearly equivalent to 1190 €, 2025 prices). The total amount of compensation paid within each category ranged from 4552 € to 2,851,309 € (Table 3). Among patients receiving financial compensation, the smallest amount paid was 339 € and the highest 595,470 € (median 9382 €).

Of the 513 resolved claims, 147 (29%) were appealed. Of these, 28 were still pending at the time of analysis. In 114 appeals, the decision was unchanged. In five appeals (4%), the original decision was overruled, with two decisions changed from approved to rejected (one appealed by the chiropractor, one by the patient), two from rejected to approved (both appealed by the patient), and one originally rejected was again rejected but on a new basis (appealed by the patient).

CAD was involved in 51 claims (10%), of which a decision had been made in 48 of the claims at the time of the study. The three most frequently noted presenting ICPC-2 diagnostic codes were *L83: Cervical syndrome* (n = 19, 37%), *L01: Symptom/complaint from the cervical region* (n = 9, 18%), and *L84: Back syndrome without radiating pain* (n = 7, 14%). Other ICPC–2 diagnostic codes were *L95: Tension headache* (n = 5), or *L02: Symptom/complaint from the thoracic region*, *L03: Symptom/complaint from the lumbar region*, *A98: Health maintenance/preventive medicine, L19: Muscle symptom/complaint NOS, L91: Osteoarthrosis, other, L08: Shoulder symptom/complaint,* and *N80: Head injury, other*, all n ≤ 3. Injury to the vertebral artery was most common (33%), with 13 out of 29 resolved claims being approved for compensation (45%) (Table 4). Approved claims had onset of CAD symptoms within 2 days of consultation, and nearly all (93%) had neck pain as the presenting complaint. Over 10 years, the rate of approved CAD claims (n = 14) was ~ 0.7 per million consultations.

**Table 4:** Overview of CAD claims

	Number of claims		Approved		Rejected		Pending	
Type of CAD	n	(%)	n	(%)	n	(%)	n	(%)
Injury, vertebral artery	32	(62.7)	13	(40.6)	16	(50.0)	3	(9.4)
Injury, carotid artery	13	(25.5)	0	(0.0)	13	(100)	0	(0)
Other (bleeding. thrombus etc.)	6	(11.8)	1	(16.7)	5	(83.3)	0	(0)
Total	51	(100)	14	(27.5)	34	(66.7)	3	(5.9)
*Time from consultation to onset of symptoms*								
During consultation or right after	16	(31.4)	9	(56.3)	7	(43.8)	0	(0.0)
< 1 day	11	(21.6)	4	(36.4)	6	(54.5)	1	(9.1)
≤ 2 days	4	(7.8)	1	(25.0)	3	(75.0)	0	(0.0)
2–7 days	6	(11.8)	0	(0.0)	5	(83.3)	1	(16.7)
≥ 7 days	11	(21.6)	0	(0.0)	10	(90.9)	1	(9.1)
Unclear timing based on data	2	(3.9)	0	(0.0)	2	(100)	0	(0.0)
Symptoms before consultation	1	(2.0)	0	(0.0)	1	(100.0)	0	(0.0)
*Characteristics of cases (n = 49)*								
Neck pain prior to consultation	46	(93.9)	13	(92.9)	33	(71.7)	–	–
Headache prior to consultation	24	(49.0)	11	(78.6)	13	(54.2)	–	–
Neurologic symptoms prior to consultations*	15	(30.6)	5	(35.7)	10	(66.7)	–	–
Cervical treatment with manipulation and mobilization	47	(95.9)	13	(92.9)	34	(72.3)	–	–

^*^Includes sensory disturbances, muscle weakness, radiating pain, or cranial nerve/reflex abnormalities without another known cause

There was an equal distribution of men and women among CAD claimants and among those with approved claims (Table 5). The median age of women in both groups was approximately 10 years younger than men.

**Table 5:** Age and gender distribution of CAD claimants

	All CAD claimants			Approved		
	Total (n = 51)	Men n = 26	Women n = 25	Total n = 14	Men n = 7	Women n = 7
Median age, years (IQR)	41 (34.5; 49.5)	46 (41.0; 52.0)	37 (33.0; 41.0)	38 (35; 46.5)	47 (44.5; 59.0)	35 (35.0; 37.5)

## Discussion

Between 2013 and 2022, the Danish Patient Compensation Association received 519 claims related to chiropractic consultations, corresponding to approximately 52 claims per year, or an average of 2.6 claims per 100,000 consultations. The number of complaints per 100,000 consultations per year more than doubled between 2013 and 2022. Only 32 claims were approved, representing 6% of all claims, with a total compensation payout of 3.0 million €. The majority of claims pertained to treatment side effects, exacerbation of symptoms, or diagnostic errors. For CAD, the approval rate was 29%. All approved CAD claims had symptom onset within two days of the chiropractic consultation and were categorized under the fairness rule. Claims concerning CAD constituted 10% of all claims and accounted for 94% of the total amount of compensation paid. The relatively high number of CAD–related claims may be caused by the fact that the most common presenting clinical features of CAD (neck pain and headache) [39] can mimic musculoskeletal disorders commonly treated in chiropractic practice. However, they might also reflect a greater awareness of CAD in connection with chiropractic care among patients and other health professionals. Nonetheless, only one third of claims were approved for compensation.

When comparing across professional groups in the Danish healthcare sector, the number of compensation claims for chiropractors was lower than claims for general practitioners (3.7 vs. 2.6 per 100,000 consultations), but higher than for physio- and occupational therapists (0.78 vs. 2.6 per 100,000 consultations) [31]. The comparatively low number of claims among physiotherapists cannot be fully explained and deserves further investigation. Potential contributing factors may include differences in patient populations and scope of practice, for example physiotherapists being more involved in broader rehabilitation settings such as neurorehabilitation or cardiovascular rehabilitation. In addition, claims concerning physiotherapists and occupational therapists are administratively grouped together within the Danish Patient Compensation Association, precluding profession–specific estimates for physiotherapists in private practice.

The approval rate for compensation claims was significantly lower for chiropractors, with only 6.2% of claims being recognized compared to 34% of claims involving general practitioners and 16.0% of claims involving physio- and occupational therapists. However, the median compensation amount paid was higher (9,382 €) in claims involving chiropractors compared to general practitioners (3,333 €) and physio- and occupational therapists (7,516 €), indicating a higher degree of severity and consequences in the chiropractic claims. This aligns with the largest amounts being paid to patients with CAD.

When comparing to international settings, the number of claims in Denmark is considerably lower than those observed among British general practitioners (8 injury reports per 10,000 consultations), [1] most likely being reflective of different compensation systems, terms, and conditions.

Compared to complaints registered for Danish chiropractors between 2004 and 2013, a doubling is observed in the total number of compensation claims and the number of claims per 100,000 consultations. However, the approval rate was 50% lower, suggesting an increased number of non-compensable claims [9]. In 2012, a report from the UK’s General Medical Council noted a pattern of annual increases in the number of patient complaints [40]. They suggested that higher patient expectations for treatment and a general increase in the willingness to file complaints played key roles [40]. Additionally, the Danish Patient Compensation Association has continuously worked to raise awareness of the compensation scheme—among patients, healthcare professionals, and even on social media—to ensure that the appropriate injuries are reported and compensated [41, 42]. In addition, after publication of Jevne's 2014 paper, Danish chiropractors heavily debated the findings, and there was a general increased awareness of reporting potential compensable injuries and patients’ right to compensation (which healthcare professionals in Denmark, as mentioned, by law are obliged to inform the patient about) [27]. Thus, it can be assumed that this has increased chiropractors' inclination to report on behalf of the patient or to make patients aware of the possibility of seeking compensation if injuries occur during treatment.

Outcomes and side effects of low and medium severity were the most common reasons for filing a compensation claim, similar to findings among Dutch general practitioners [6]. Since 2013, no compensation claims in this category have been approved. All rejections were based on the exacerbation of patients’ symptoms being caused by the natural history of the patient’s underlying condition and not an injury. These types of complaints could potentially be mitigated by offering patients better information about, first and foremost, the natural course of the patient’s condition and expected side effects of treatment, and second, the compensational terms and conditions.

Of the total compensation paid, 94% (2.9 million €) was allocated to 14 CAD claims. The patients' median age was 38 years, but differed by approximately 10 years between men and women. This aligns with existing research, suggesting that the incidence of CAD is highest in patients aged 30–50 years, [13, 43, 44] with women being younger than men [44, 45]. The amounts paid to this group were due to the permanent disabilities and lost income experienced by patients of working age. In all the approved CAD claims, symptoms appeared two days or less after the consultation, and in 13 out of 14 claims, the vertebral artery was involved, indicating emphasis on the timing of events and anatomical plausibility in the ruling. All 14 claims were approved under the fairness rule, stating that even though the chiropractor had practiced according to current standards, the patient was still in a worse condition after the consultation than what could reasonably have been expected, thus exempting the chiropractor from any wrongdoing.

Strengths of our study include nationally near-complete data and the use of HCAT, which is an internationally recognized analysis tool that enables systematic, reliable, and comparable data collection across settings and countries [33–35]. Standardized training in using the tool and pilot testing further increased the reliability of the data extraction. Replication of the data collection from the study by Jevne et al. [9] enabled comparison and detection of trends in claims over time.

We have conducted a descriptive study of patient compensation claims. While it may provide a transparent overview of trends related to patients’ experiences, the injuries approved and compensated, providing a health learning basis for quality improvement, it cannot establish causality between consultations and injuries—or treatments and injuries. Furthermore, our study does not include chiropractor- or clinic-level data due to the European General Data Protection Regulation (GDPR) restrictions. Since the number of practicing chiropractors in Denmark is relatively small, providing data at this level of granularity would risk making individual chiropractors or patients identifiable.

Data for this study were drawn from consultations of the approximately 90% of all Danish chiropractors who work within the national reimbursement framework. Thus, there is an underreporting of consultations by an estimated 10%. Since patients visiting chiropractors outside the collective agreement are equally entitled to seek compensation, the number of claims per 100,000 consultations is likely slightly overestimated.

The number of compensation claims could likely be significantly reduced with increased focus among chiropractors on informing patients about the natural course of their conditions and the common but benign side effects following chiropractic treatment. Despite highlighting this issue in 2014 following publication of the Jevne study [9], and the subsequent awareness raised through continuing education and debates on the topic, we still see a high and even increasing number of non-compensable claims. Therefore, future continuing education and discussions should continue to focus on effective communication with patients and other healthcare professionals about what to expect after treatment from a chiropractor, as well as an emphasis on identifying which symptoms, experiences, and injuries actually trigger compensation, so that this information can be communicated to patients. By doing so, administrative work for patients, chiropractors, physicians, and the Danish Patient Compensation Association could likely be reduced, and significant amounts of work time and resources could be saved for all parties involved. Finally, monitoring developments in patient complains and injuries following encounters with the health systems—including with chiropractic care – can inform delivery of healthcare, communication with patients and the public, and development of good and fair compensation systems.

## Conclusion

Injury claims related to chiropractic care in Denmark increased between 2013 and 2022, but approval rates remained low. Most claims concerned dissatisfaction with treatment outcome or worsening of symptoms. Cervical artery dissection-related claims had the highest approval rate and accounted for the highest compensation. When approved, they were compensated based on the fairness rule stating that the chiropractor was not blamed for the injury. Better communication between patients and chiropractors about expectations for treatment, the natural course of conditions, and expected reactions to treatment will likely reduce the number of claims significantly.

## Supplementary Information

Below is the link to the electronic supplementary material.

Supplementary Material 1

Supplementary Material 2

## Data Availability

The data is available from the Danish Patient Compensation Association on reasonable request.

## Abbreviations

CAD: Cervical artery dissection
GDPR: General data protection regulation
HCAT: Healthcare complaint analysis tool
