# Supplementary material for: Compensation claims for chiropractic in Denmark 2013–2022
Source: Chiropr Man Therap. 2026 Feb 24;34:5. doi: 10.1186/s12998-026-00627-1 (PMC12931014; doi:10.1186/s12998-026-00627-1)
Supplement: Supplementary file 1 — Supplementary Material 1 [file 12998_2026_627_MOESM1_ESM.docx]

Supplementary File 1. Extracted variables and their definitions

| **Variable** | **Definitions and categories** |
| --- | --- |
| Claim Number | Unique identifier for each claim |
| Date of Claim Registration | Date when the claim letter was officially registered |
| Patient's Date of Birth | Date of birth of the patient |
| Patient's Gender | 1: Male 2: Female 3: Unknown/Other |
| Who Submitted the Claim? | 1: Relative 2: Patient 3: Unknown/Other (Specify) |
| Date of Incident | The date on which the alleged injury occurred |
| Patient's Age at Time of Injury | Age of the patient when the injury occurred |
| Decision Year† | Year of decision (rejected/approved) |
| Closure Year | Year when the claim was officially closed |
| Appeal Status (Date) | Date of appeal submission, if applicable |
| Decision Outcome† | 1: Rejected 2: Undecided 3: Approved |
| Decision Code | Text field specifying the decision rationale |
| Claim Status | 1: Closed 2: Ongoing |
| Compensation Amount† | Text field specifying the awarded compensation sum |
| Claim Appealed? † | 1: Yes 2: No |
| Problem Category* | 1: Quality 2: Safety 3: Environment 4: Organizational Processes 5: Responsiveness 6: Communication 7: Respect and Patient Rights |
| Subcategories – Quality | 1: Neglect – hygiene & personal care 2: Neglect – nutrition & hydration 3: General neglect 4: Rough handling & discomfort 5: Examination & monitoring 6: Treatment planning & follow-up 7: Treatment outcome |
| Subcategories – Safety | 1: Diagnostic error 2: Medication error 3: General error 4: Lack of response 5: Clinical skills 6: Collaboration 88: Other (Specify) |
| Subcategories – Environment | 1: Accommodation 2: Preparedness 3: Ward cleanliness 4: Equipment 5: Staffing 6: Safety 7: Infection control 88: Other (Specify) |
| Subcategories – Organizational Processes | 1: Delay – accessibility 2: Delay – procedures 3: Transitions 4: General delay 5: Bureaucracy 6: Appointments 7: Documentation 8: Continuity 9: Prioritization 88: Other (Specify) |
| Subcategories – Responsiveness | 1: Ignoring patients 2: Dismissing patients 3: Feigned responsiveness 88: Other (Specify) |
| Subcategories – Communication | 1: Delayed information 2: Incorrect information 3: Lack of information 4: Failure to involve relatives 5: Language barriers 88: Other (Specify) |
| Subcategories – Respect and Patient Rights | 1: Lack of respect 2: Confidentiality 3: Rights – guarantee 4: Rights 5: Consent 6: Privacy 88: Other (Specify) |
| Severity Level* | 1: Low 2: Medium 3: High |
| Personnel Involved* | 1: Administration 2: Doctors 3: Nursing staff (not included) 4: Other (Specify) 5: Chiropractor 6: Massage therapist 7: Physiotherapist |
| Geographical Region of Claim | 1: Capital Region 2: Central Denmark Region 3: North Denmark Region 4: Zealand Region 5: Southern Denmark Region 6: Unknown |
| Primary Sector Treatment Stage* | 1: Access to treatment 2: During visit to clinic 3: During consultation/treatment 4: Referral/follow-up 5: Unspecified or other (Specify) |
| Claim Letter Excerpt* | Text field capturing direct excerpts from claim letters. |
| Reasons Claim Cannot Be Coded* | 1: Not a claim 2: Claim letter illegible 3: Already coded 4: Missing information 88: Other |
| Primary Diagnosis | Three text fields for primary diagnosis 1, 2, and 3 noted by the Danish Patient Compensation Association |
| Severity of Patient-Reported Injury* | 1: Insignificant 2: Minor 3: Moderate 4: Significant 5: Catastrophic (Specify) |
| Symptom Diagnosis | ICPC-2 diagnostic code based on symptom classification (e.g., cervical pain, dizziness, muscle pain) noted by the chiropractor |
| Treatment Modality Used | 1: Manipulation therapy 2: Soft tissue treatment 3: Dry needling 4: Patient education 5: Exercises 6: None 88: Other (Specify) |
| Classification by Jevne's Complaint Categories† | 1: Symptom worsening 2: Alleged disc herniation 3: Delayed referral 4: New symptoms/injury post-treatment 5: Treatment-induced fracture 6: CAD 7: Rib injury 8: Accidents 9: Miscellaneous (e.g., nerve/organ injuries) |

**HCAT variables; † Jevne et al. [1]. Abbreviations: Cervical artery dissection (CAD)*
