# Supplementary material for: Compensation claims for chiropractic in Denmark 2013–2022
Source: Chiropr Man Therap. 2026 Feb 24;34:5. doi: 10.1186/s12998-026-00627-1 (PMC12931014; doi:10.1186/s12998-026-00627-1)
Supplement: Supplementary file 2 — Supplementary Material 2 [file 12998_2026_627_MOESM2_ESM.docx]

**Supplementary File 2.** Variables relating to cervical artery dissection

| **Variable** | **Definition and categories** |
| --- | --- |
| Characteristics of Clinical Course | 1: Neck pain (yes/no) 2: Headache (yes/no) 3: Neurological deficits* (yes/no) 4: Cervical treatment with manipulation or mobilisation |
| **Type of Vascular Injury Diagnosed** | 1: Injury to the vertebral artery 2: Injury to the carotid artery 3: Other (bleeding, thrombus, etc.) with or without radiological evidence |
| Time from Treatment to Symptom Onset | 1: Immediately after or during treatment  2: < 1 day  3: 1–2 days  4: 3–6 days  5: ≥ 7 days  6: Unclear based on available data  7: Symptoms present before treatment |

*Includes sensory disturbances, muscle weakness, radiating pain, or cranial nerve/reflex abnormalities without another known cause
